# A Review on the Role of SPRY4-IT1 in the Carcinogenesis

**DOI:** 10.3389/fonc.2021.779483

**Published:** 2022-01-13

**Authors:** Soudeh Ghafouri-Fard, Tayyebeh Khoshbakht, Mohammad Taheri, Seyedpouzhia Shojaei

**Affiliations:** ^1^ Department of Medical Genetics, School of Medicine, Shahid Beheshti University of Medical Sciences, Tehran, Iran; ^2^ Men’s Health and Reproductive Health Research Center, Shahid Beheshti University of Medical Sciences, Tehran, Iran; ^3^ Skull Base Research Center, Loghman Hakim Hospital, Shahid Beheshti University of Medical Sciences, Tehran, Iran; ^4^ Department of Critical Care Medicine, Imam Hossein Medical and Educational Center, Shahid Beheshti University of Medical Sciences, Tehran, Iran

**Keywords:** SPRY4-IT1, cancer, biomarker, expression, carcinogenesis

## Abstract

Sprouty RTK signaling antagonist 4-intronic transcript 1 (SPRY4-IT1) is a long non-coding RNA (lncRNA) encoded by a gene located on 5q31.3. This lncRNA has a possible role in the regulation of cell growth, proliferation, and apoptosis. Moreover, since SPRY4-IT1 controls levels of lipin 2, it is also involved in the biosynthesis of lipids. During the process of biogenesis, SPRY4-IT1 is produced as a primary transcript which is then cleaved to generate a mature transcript which is localized in the cytoplasm. SPRY4-IT1 has oncogenic roles in diverse tissues. A possible route of participation of SPRY4-IT1 in the carcinogenesis is through sequestering miRNAs such as miR-101-3p, miR‐6882‐3p and miR-22-3p. The sponging effect of SPRY4-IT1 on miR-101 has been verified in colorectal cancer, osteosarcoma, cervical cancer, bladder cancer, gastric cancer and cholangiocarcinoma. SPRY4-IT1 has functional interactions with HIF-1α, NF-κB/p65, AMPK, ZEB1, MAPK and PI3K/Akt signaling. We explain the role of SPRY4-IT1 in the carcinogenesis according to evidence obtained from cell lines, xenograft models and clinical studies.

## Introduction

SPRY4 Intronic Transcript 1 (SPRY4-IT1) is a long non-coding RNA (lncRNA). This transcript is encoded by a gene on the cytogenetic band 5q31.3. During the process of biogenesis, SPRY4-IT1 is produced as a primary transcript which is then cleaved to generate a mature transcript which is localized in the cytoplasm (1). Since the complete size and structure of the primary and cleaved transcripts of SPRY4-IT1 are not clear, it has been speculated that the primary transcript is an alternatively spliced variant of SPRY4 (https://www.ncbi.nlm.nih.gov/gene/100642175).

A pioneer study in this field has suggested that SPRY4-IT1 is originated from an intronic region of the *SPRY4* gene. *In silico* studies have predicted that SPRY4-IT1 has numerous long hairpins in its secondary configuration. Based on the results of RNA-FISH experiments in the melanoma cells, SPRY4-IT1 is mainly localized in the cytoplasm. Since SPRY4-IT1 silencing has altered growth, differentiation, and apoptosis in melanoma cells, it has been suggested that SPRY4-IT1 has a role in the etiology of melanoma (2). Subsequent studies have provided further evidence for participation of SPRY4-IT1 in other types of cancers as well. In normal cells, this lncRNA can regulate cell cycle progression and cell proliferation. In the current review, we explain the role of SPRY4-IT1 in the carcinogenesis based on evidence obtained from cell lines, xenograft models and clinical studies.

## Cell Line Studies

SPRY4-IT1 has been found to up-regulated in colorectal cancer cells. SPRY4-IT1 regulates growth and glycolysis of these cells through enhancing expression of PDK1. SPRY4-IT1 has affected glucose intake, lactic acid synthesis, and levels of ATP in colorectal cancer cells (3). SPRY4-IT1 has also been demonstrated to increase proliferation, migratory potential and invasiveness of colorectal cancer cells. Most notably, SPRY4-IT1 enhances expression of epithelial-mesenchymal transition (EMT)-associated genes. Mechanistically, SPRY4-IT1 negatively regulates expression of miR-101-3p in these cells through binding with this miRNA (4). SPRY4-IT1 up-regulation in a colorectal cancer cell line has resulted in differential expression of several genes among them has been TCEB1. This transcription elongation factor subunit can interact with the Alu element in the 3′untranslated region (UTR) of SPRY4-IT1. Besides, SPRY4-IT1 binds with STAU1 to increase STAU1 recruitment to the 3′-UTR of TCEB1 transcript. It subsequently modulates stability and expression of TCEB1, leading to up-regulation of HIF-1α. STAU1 is attributed to the family of double-stranded RNA-binding proteins. It participates in the transport of transcripts to various subcellular localizations. Expression of SPRY4-IT1 is also activated by NF-κB/p65 (5).

**Figure 1:**
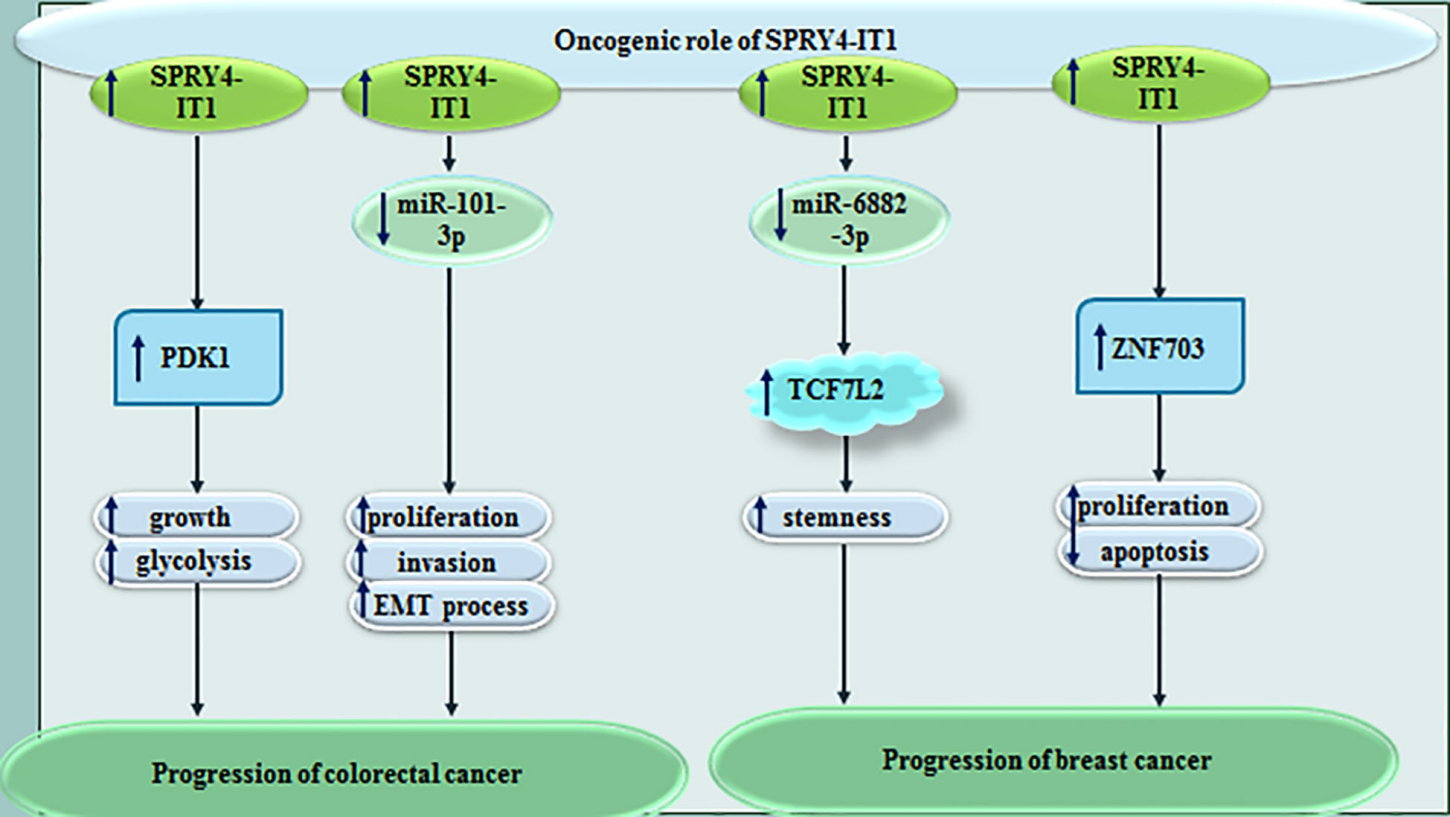
Oncogenic effect of SPRY4-IT1 in colorectal and breast cancers.

SPRY4-IT1 has also been reported to be over-expressed in MCF-7 cancer stem cells compared with MCF-7 cells. Up-regulation of SPRY4-IT1 has enhanced proliferation and stemness of breast cancer cells. Moreover, SPRY4-IT1 silencing has inhibited renewal capacity of breast cancer stem cells and maintenance of their stemness. Mechanistically, SPRY4-IT1 acts as a sponge for miR-6882-3p to affect expression of TCF7L2 (6). SPRY4-IT1 silencing in breast cancer cells has significantly inhibited their proliferation and prompted cell apoptosis. ZNF703 has been found to be a target of SPRY4-IT1 in these cells (7). The encoded protein by this gene is involved in nucleic acid binding and DNA-binding transcription factor binding. **Figure 1** shows the oncogenic effect of SPRY4-IT1 in colorectal and breast cancers.

**Figure 2:**
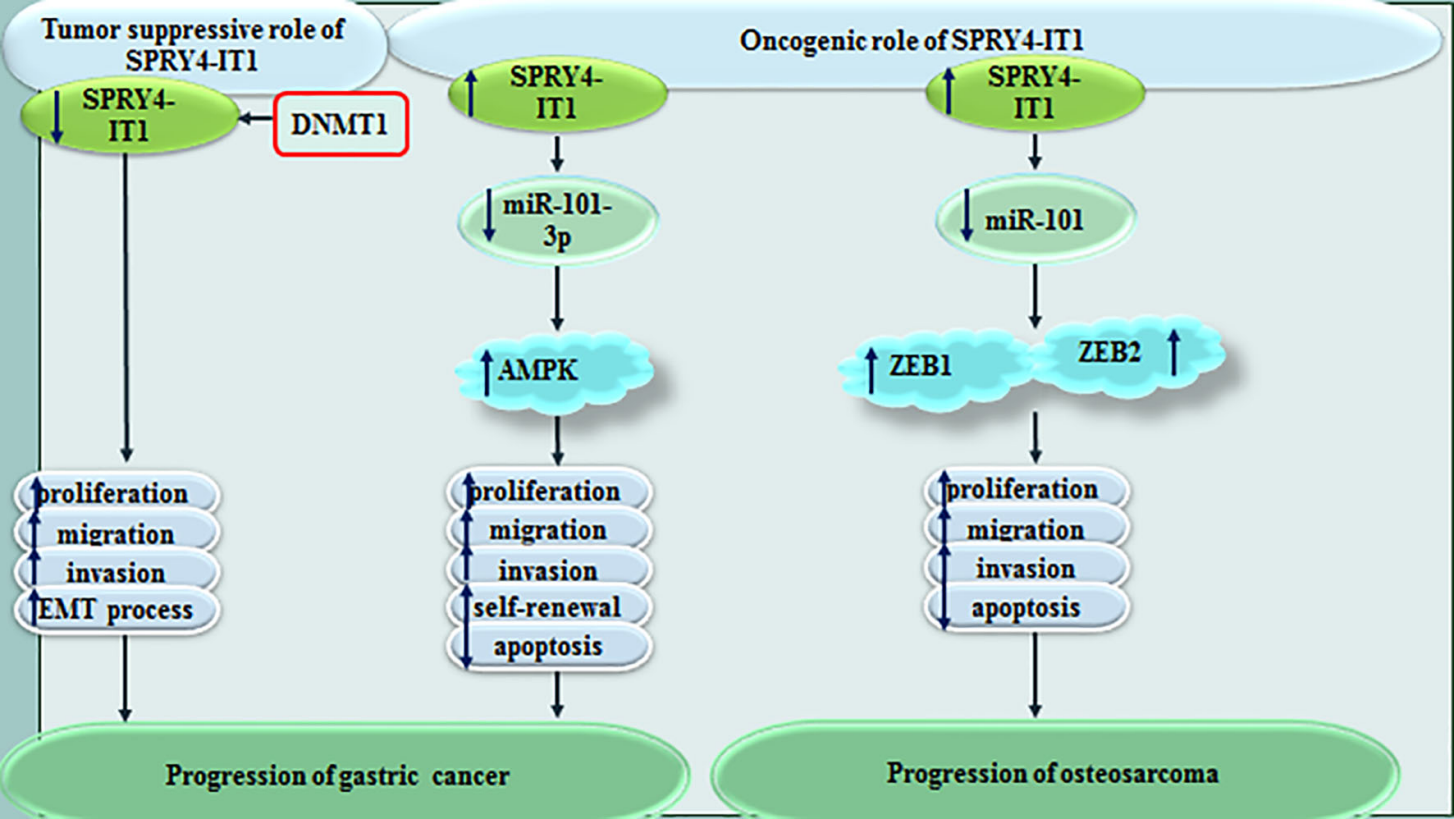
Effect of SPRY4-IT1 in the pathogenesis of gastric cancer and osteosarcoma.

Cao et al. has shown that SPRY4-IT1 silencing significantly constrains proliferation of gastric cancer cells through inducing G1 arrest and enhancing apoptosis. SPRY4-IT1 acts as a sponge for miR-101-3p to increase expression of AMPK (8). On the other hand, Xie et al. have shown tumor suppressor role of SPRY4-IT1 in gastric cancer. DNA methylation has been found to be the main mechanism of control of SPRY4-IT1 expression in these cells. Besides, SPRY4-IT1 has been shown to affect EMT in gastric cancer cells (9). In osteosarcoma cells, SPRY4−IT1 has been shown to promote cancer progression through sequestering miR-101 and enhancing expressions of ZEB1 and ZEB2 (10). **Figure 2** shows the effect of SPRY4-IT1 in the pathogenesis of gastric cancer and osteosarcoma.

In lung cancer, SPRY4-IT1 has been shown to reverses resistance to cisplatin through decreasing expression of MPZL-1 and suppression of EMT process (11). MPZL-1 is functionally related with tyrosine kinases/adaptors and adhesion. Moreover, EZH2-related epigenetic down-regulation of SPRY4-IT1 has promoted proliferation and metastatic ability of lung cancer cells through influencing EMT (12). Contrary to these studies, Zhang et al. have stated that SPRY4-IT1 increases migration and invasiveness of lung adenocarcinoma cells (13).

**Figure 3:**
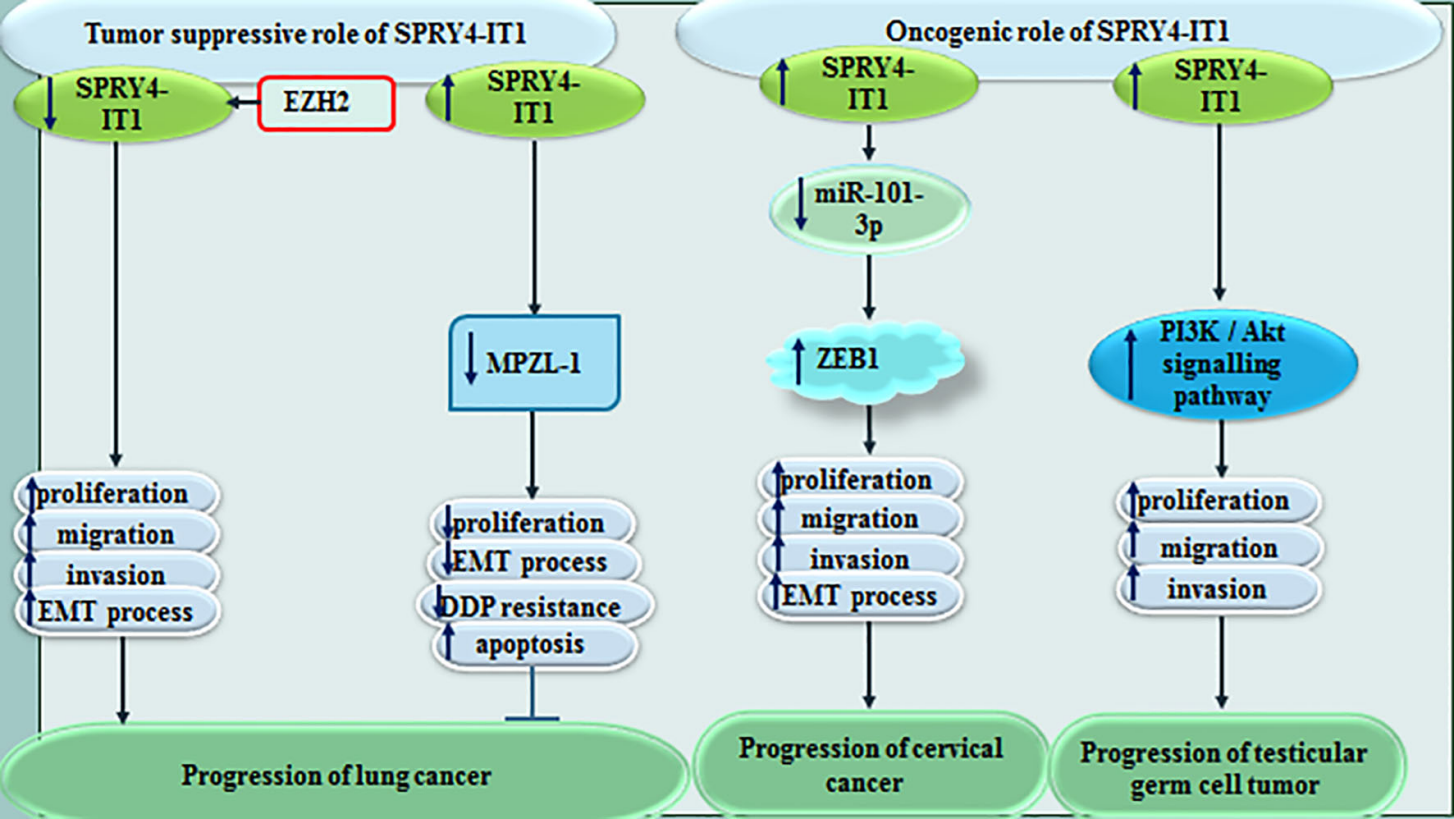
Effect of SPRY4-IT1 in the pathogenesis of lung, cervical and testicular cancers.

In cervical cancer, SPRY4-IT1 can increase EMT influencing activity of the miR-101-3p/ZEB1 axis (14). In testicular germ cell tumors, SPRY4-IT1 has been found to suppress growth of cancer cells and phosphorylation of Akt (15). **Figure 3** shows the role of SPRY4-IT1 in the pathogenesis of lung, cervical and testicular cancers.

**Figure 4:**
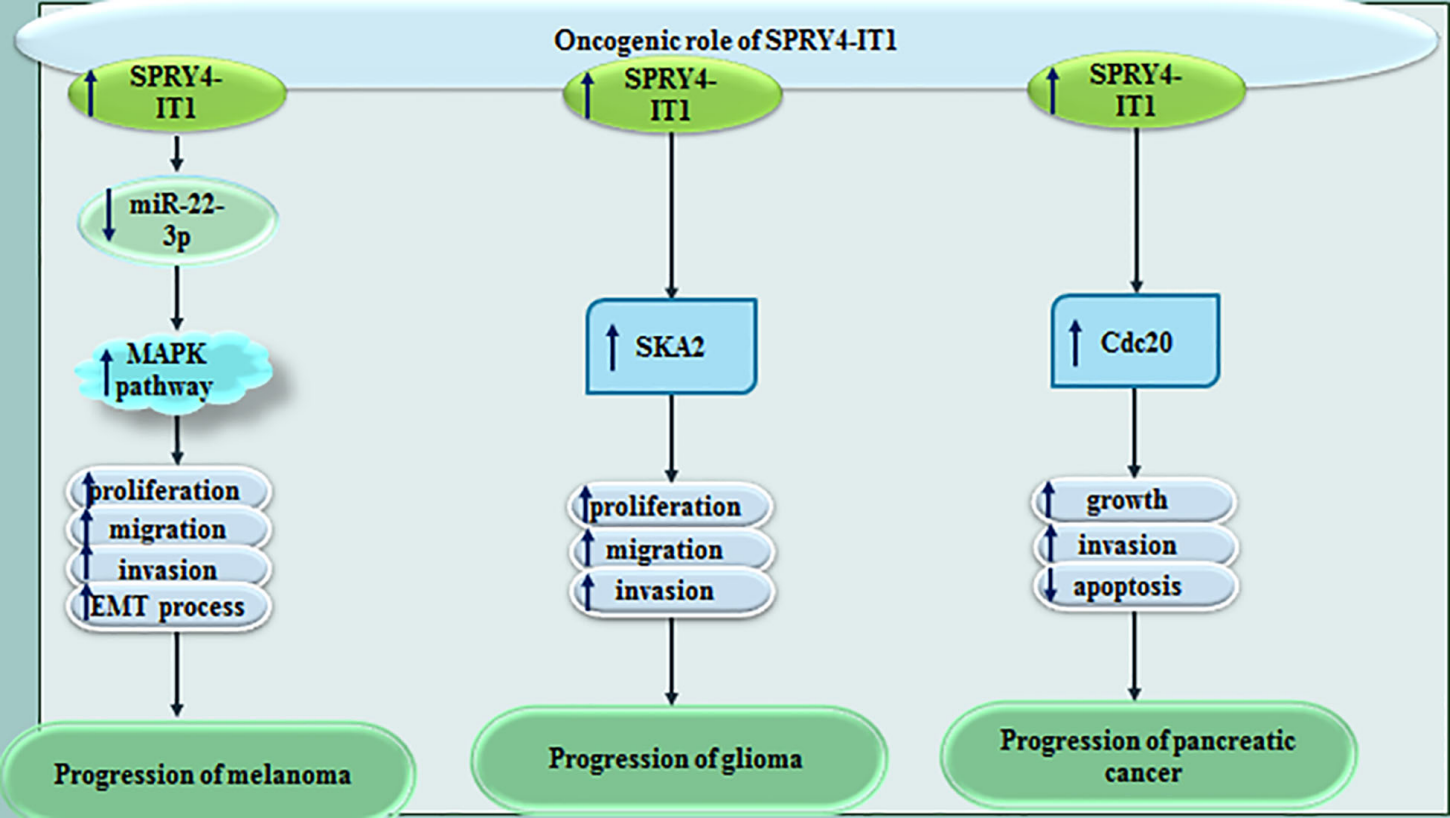
Oncogenic role of SPRY4-IT1 in melanoma, glioma and pancreatic cancer.

SPRY4-IT1 levels have been found to be elevated in melanoma cells lines when compared to the normal skin cell line. Up-regulation of this lncRNA has been attended by down-regulation of miR-22-3p. Dual luciferase reporter assay has confirmed the interaction between SPRY4-IT1 and miR-22-3p. Under-expression of SPRY4-IT1 has blocked proliferation, invasiveness, migration, and EMT of melanoma cells. Over-expression of miR-22-3p has been shown to decelerate phosphorylation of p38MAPK, MAPKAPK and Hsp27, thus miR-22-3p decreases activity of the p38MAPK/MAPKAPK/Hsp27 signaling (16). In glioma, SPRY4-IT1 has been revealed to stimulate cell proliferation and invasion *via* up-regulating SKA2 (17). It has a role in enhancement of EMT of glioma cells as well (18). Moreover, SPRY4-IT1 enhances proliferation and invasiveness of pancreatic cancer cells through regulation of Cdc20 (19). **Figure 4** shows oncogenic role of SPRY4-IT1 in melanoma, glioma and pancreatic cancer.

**Figure 5:**
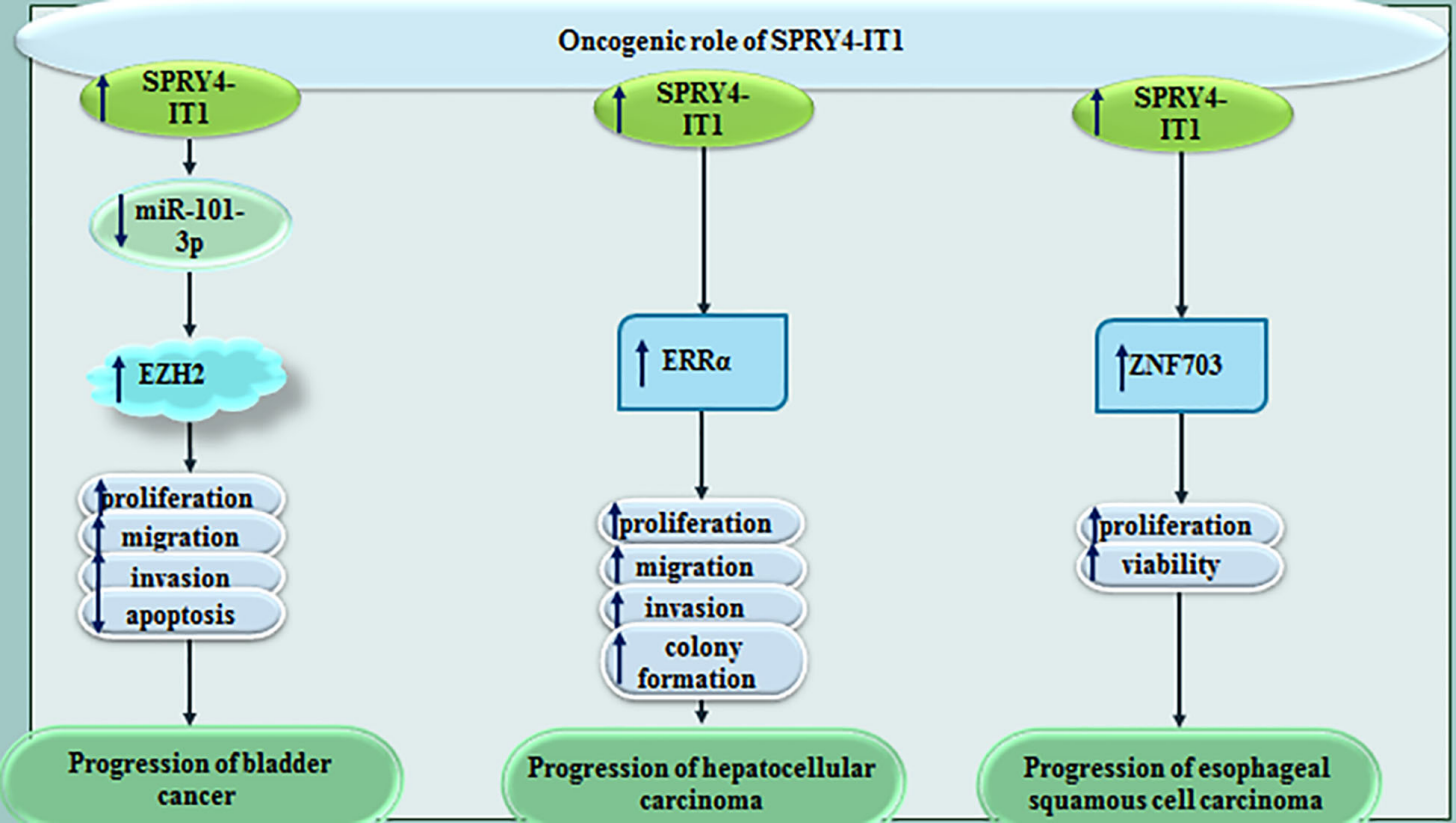
Oncogenic role of SPRY4-IT1 in bladder, liver and esophageal cancers.

In bladder cancer cells, SPRY4-IT1 sequesters miR-101-3p to increase proliferation and metastatic ability of neoplastic cells *via* enhancing expression of EZH2 (20). In hepatocellular carcinoma cells, SPRY4-IT1 silencing has attenuated cell proliferation, colony formation, invasiveness and migratory potential. SPRY4-IT1 silencing has led to cell cycle arrest at G0/G1 stage and stimulated cell apoptosis. Moreover, SPRY4-IT1 silencing has inhibited expression of estrogen-related receptor α (ERRα) at transcript and protein level (21). Upregulation of SPRY4-IT1 has also been shown to increase viability of esophageal squamous cell carcinoma cells through inducing expression of zinc finger 703 (22). **Figure 5** shows impact of SPRY4-IT1 in the pathoetiology of bladder, liver and esophageal cancers.

**Table 1** summarizes the effect of SPRY4-IT1 in cancers based on cell line studies.

**Table 1:** Effect of SPRY4-IT1 in cancers based on cell line studies.

Tumor type	Targets/Regulators and Signaling Pathways	Cell line	Function	Reference
Colorectal cancer	PDK1	NCM460, T84, HT-29, SW480	Δ SPRY4-IT1: ↓ growth, ↓ viability, ↓ colony formation, ↓ glycolysis	(3)
	_	HCT116, LoVo, RKO, SW620, SW480, 293T	Δ SPRY4-IT1: ↓ proliferation, ↓ growth, ↓ invasion, ↓ EMT process, ↑ G0/G1 phase arrest, ↑ apoptosis	(23)
	_	HT-29, HCT-116, and SW-480, FHC	Δ SPRY4-IT1: ↓ proliferation, ↓ migration, ↓ invasion, ↓ EMT process, ↑ G0/G1 phase arrest	(24)
	miR-101-3p	LoVo, RKO, SW620, and SW480	Δ SPRY4-IT1: ↓ proliferation, ↓ invasion, ↓ EMT process	(4)
	TCEB1, HIF-1α signaling pathways, NF-κB/p65	HCT 116, Caco-2, HT-29, SW480, SW620	↑ SPRY4-IT1: ↑ migration, ↑ invasion	(5)
Breast cancer	miR‐6882‐3p, TCF7L2	MCF‐7, T47D	Δ SPRY4-IT1: ↓ stemness	(6)
	SDF-1α/CXCR4 axis, NT21MP, SKA2	SKBR-3, MCF-7, MDA-MB-231	Δ SPRY4-IT1: ↓ proliferation, ↓ migration, ↓ invasion, ↑ G0/G1 phase arrest, ↑ apoptosis	(25)
	ZNF703	MD-MB-231, MD-MB-435S, MCF-10A, MCF-7	Δ SPRY4-IT1: ↓ proliferation, ↑ G0/G1 phase arrest, ↑ apoptosis	(7)
	TCEB1, HIF-1α signaling pathways, NF-κB/p65	MCF-7, T-47D, MDA-MB-231	↑ SPRY4-IT1: ↑ migration, ↑ invasion	(5)
Ovarian cancer	TCEB1, HIF-1α signaling pathways, NF-κB/p65	Caov-3, SK-OV-3, HEK293T, OVCAR-3	↑ SPRY4-IT1: ↑ migration, ↑ invasion	(5)
	_	SKOV3, HO8910, ES-2, CAOV3, IOSE80	↑ SPRY4-IT1: ↓ proliferation, ↓ migration, ↓ invasion, ↓ EMT process, ↑ cell cycle arrest, ↑ apoptosis	(26)
Gastric cancer	miR-101-3p, AMPK	GES-1, MKN28, SGC7901, BGC823	Δ SPRY4-IT1: ↓ proliferation, ↓ migration, ↓ invasion, ↓ self-renewal, ↑ G0/G1 arrest, ↑ apoptosis	(8)
	DNMT1	SGC7901, BGC823, MGC803, AGS, MKN45, MKN28, HCG-27, GES-1	Δ SPRY4-IT1: ↑ proliferation, ↑ migration, ↑ invasion, ↑ EMT process ↑ SPRY4-IT1: ↓ proliferation, ↓ migration, ↓ invasion, ↓ EMT process	(9)
Osteosarcoma	miR-101, ZEB1, ZEB2	hFOB 1.19, U2OS, MG-63, Saos-2, 293	Δ SPRY4-IT1: ↓ proliferation, ↓ migration, ↓ invasion, ↑ apoptosis	(10)
	_	HOS, Saos-2, U2OS, MG-63, NHOst	Δ SPRY4-IT1: ↓ proliferation, ↓ migration, ↓ invasion, ↓ self-renewal, ↑ G0/G1 arrest, ↑ apoptosis	(27)
Lung cancer	MPZL-1	A549/DDP, A549	↑ SPRY4-IT1: ↓ proliferation, ↓ EMT process, ↓ DDP resistance, ↑ apoptosis	(11)
	_	H23, H1299, A549, SPC-A1, HLF	Δ SPRY4-IT1: ↓ migration, ↓ invasion	(13)
	EZH2	A549, SPC-A1, NCI-H1975, NCI-H1299, NCI-H1650, (SK-MES-1	↑ SPRY4-IT1: ↓ proliferation, ↓ migration, ↓ invasion, ↓ EMT process, ↑ apoptosis Δ SPRY4-IT1: ↑ migration, ↑ invasion	(12)
Cervical cancer	miR-101-3p, ZEB1	HeLa, CaSki	Δ SPRY4-IT1: ↓ proliferation, ↓ migration, ↓ invasion, ↓ EMT process	(14)
Testicular germ cell tumor	PI3K/Akt signaling pathway	NT2-D1, 833 K	Δ SPRY4-IT1: ↓ proliferation, ↓ migration, ↓ invasion	(15)
Melanoma	miR-22-3p, MAPK pathway	A375, A875, TE 353.SK	Δ SPRY4-IT1: ↓ proliferation, ↓ migration, ↓ invasion, ↓ EMT process	(16)
Glioma	SKA2	astrocytoma U251	Δ SPRY4-IT1: ↓ proliferation, ↓ migration, ↓ invasion	(17)
	_	U251, SF295, NHA	Δ SPRY4-IT1: ↓ proliferation, ↓ migration, ↓ EMT process	(18)
Pancreatic cancer	Cdc20	BxPC-3, PANC-1	Δ SPRY4-IT1: ↓ growth, ↓ migration, ↓ invasion, ↑ G0/G1 arrest, ↑ apoptosis	(19)
Pancreatic ductal adenocarcinoma	_	BxPC3, Capan-2, PANC1, SW1990	Δ SPRY4-IT1: ↓ proliferation, ↑ apoptosis	(28)
Cholangiocarcinoma	SP1, miR-101-3p, KLF2, LATS2, EZH2, LSD1, DNMT1	RBE and HCCC-9810, HIBEC, CCLP-1, HuCCT1, Huh-28, KMBC, QBC939	Δ SPRY4-IT1: ↓ proliferation, ↓ EMT process, ↑ apoptosis	(29)
Gallbladder carcinoma	_	EH-GB1, GBC-SD, SGC-996, NOZ, 293T	Δ SPRY4-IT1: ↓ proliferation, ↓ migration, ↓ EMT process, ↑ SPRY4-IT1: ↑ proliferation, ↑ migration, ↑ EMT process,	(30)
Bladder cancer	miR-101-3p, EZH2	SV-HUC-1, EJ, UMUC3, T24T	Δ SPRY4-IT1: ↓ proliferation, ↓ migration, ↓ invasion, ↑ apoptosis	(20)
	_	J82, T24, SW780, SV-40, SV-HUC-1	Δ SPRY4-IT1: ↓ proliferation, ↓ migration, ↓ invasion	(31)
Hepatocellular carcinoma	ERRα	HL7702, MHCC97L, MHCC97H, HepG2, SMMC7721	Δ SPRY4-IT1: ↓ proliferation, ↓ migration, ↓ invasion, ↓ colony formation, ↑ G0/G1 arrest, ↑ apoptosis	(21)
Esophageal squamous cell carcinoma	_	KYSE-450, KYSE-510, KYSE-150, KYSE-180, KYSE-30, KYSE-70s, and KYSE-140	Δ SPRY4-IT1: ↓ proliferation, ↓ migration, ↓ invasion	(32)
	ZNF703	TE-13	Δ SPRY4-IT1: ↓ proliferation, ↓ viability	(22)
Clear cell renal cell carcinoma	_	786-O, ACHN, Caki-1, Caki-2, HK-2	Δ SPRY4-IT1: ↓ proliferation, ↓ migration, ↓ invasion	(33)

Δ, knock-down or deletion; DDP, cisplatin.

## Animal Studies

Experiments in animal models of cancers have verified the influence of SPRY4-IT1 in the carcinogenesis. For instance, up-regulation of SPRY4-IT1 has enhanced proliferation and stemness of breast cancer cells in animal models. Besides, investigations in animal models have shown that SPRY4-IT1 silencing inhibits renewal capacity of breast cancer stem cells and reduces their stemness (6). In xenograft models of gastric cancer, two different studies have reported conflicting results. While in BALB/c nude mice, SPRY4-IT1 silencing has decreased malignant behavior of neoplastic cells (8), another study in male athymic mice has shown the reverse results (9). In animal models of lung cancer, concomitant up-regulation of SPRY4-IT1 and cisplatin treatment has attenuated tumor growth and metastasis (11). However, in other types of cancers, xenograft models have shown oncogenic roles of SPRY4-IT1 (**Table 2**).

**Table 2:** Role of SPRY4-IT1 in cancers based on animal studies.

Tumor Type	Animal models	Results	Reference
Breast cancer	3‐ to 4‐week‐old female BALB/c(nu/nu) mice Mice were divided into the four groups (n = 6 per group): NC‐cDNA with MCF‐7; SPRY4‐IT1‐cDNA with MCF‐7; sh‐NC with MCF‐7 CSCs; and sh‐SPRY4‐IT1 with MCF‐7 CSCs	↑ SPRY4-IT1: ↑ tumor size, ↑ tumor weigh, ↑ stemness, ↑ self‐renewal capacity	(6)
Gastric cancer	3‐ to 4‐week‐old male BALB/c nude mice Mice injected with BGC823 cells transfected with sh-SPRY4-IT1 or sh-NC	Δ SPRY4-IT1: ↓ tumor weight, ↓ tumor growth, ↓ tumor size	(8)
	5 weeks female athymic BALB/c nude mice Mice injected with BGC-823 cells transfected with pCDNA-SPRY4-IT1 or empty vector	↑ SPRY4-IT1: ↓ tumor weight, ↓ tumor size, ↓ metastasis	(9)
Osteosarcoma	BALB/c nude mice 24 nude mice were divided into 4 groups (n=6/group); MG-63/shNC (control), MG-63/shSPRY4-IT1 (treatment), U2OS/shNC (control) and U2OS/shSPRY4-IT1 (treatment). Mice were injected with MG-63 or U2OS cells transfected with shNC or shSPRY4-IT1.	Δ SPRY4-IT1: ↓ tumor volume, ↓ tumor weight	(10)
Lung cancer	4-week old female athymic BALB/c nude mice 10 Mice (n = 5 per group) were injected with A549/DDP cells transfected with pCDNA-SPRY4-IT1 and empty vector.	↑ SPRY4-IT1 + DDP Treatment: ↓ tumor volume, ↓ tumor weight	(11)
	4-week old female athymic BALB/c nude mice Mice were injected with SPC-A1 cells transfected with pCDNA-SPRY4-IT1 and empty vector. 4-week old male athymic mice 9 mice were injected with A549 cells transfected with pCDNA-SPRY4-IT1 or empty vector.	↑ SPRY4-IT1: ↓ tumor volume, ↓ tumor weight, ↓ metastasis	(12)
Cervical cancer	4-week old female BALB/c nude mice Mice (*n*=6 per group) were injected with HeLa and CaSki cells transfected with *SPRY4-IT1* shRNA or negative control.	Δ SPRY4-IT1: ↓ tumor volume, ↓ tumor weight, ↓ metastasis	(14)
Pancreatic ductal adenocarcinoma	6-week old female nude mice Mice (n=4 per group) were injected with PANC1 cells transfected with control shRNA or SPRY4-IT1 shRNA.	Δ SPRY4-IT1: ↓ tumor weight	(28)
Cholangiocarcinoma	6-week old female BALB/c nude mice Mice (n=6 per group) were injected with HuCCT1 cells transfected with shSPRY4-IT1 or the scrambled control.	Δ SPRY4-IT1: ↓ tumor weight, ↓ tumor growth	(29)
Bladder cancer	4-week old female BALB/c nude mice Mice (n=6 per group) were injected with T24T cells transfected with SPRY4-IT1 shRNA or negative control.	Δ SPRY4-IT1: ↓ tumor volume, ↓ tumor weight	(20)
Esophageal squamous cell carcinoma	4-week old male BALB/c nude mice Mice (n=5 per group) were injected with KYSE-30 cells transfected with si-SPRY4-IT1 or si-NC.	Δ SPRY4-IT1: ↓ tumor weight, ↓ tumor growth	(32)

Δ, knock-down or deletion.

## Human Studies

Using a panel of colon, breast, and ovarian cancer tissues, Zhao et al. have found that elevation of SPRY4-IT1 expression is associated with aggressive behavior and poor clinical outcome of patients (5). Another study has shown that SPRY4-IT1 overexpression in breast cancer tissues is associated with a larger neoplasm bulk and higher pathological stage (7).

SPRY4-IT1 has also been reported to be increased in gastric cancer tissues and serum exosomes. Notably, up-regulation of SPRY4-IT1 in serum exosomes has been correlated with metastatic ability of this cancer (8). On the other hand, Xie et al. have reported down-regulation of SPRY4-IT1 in gastric cancer tissues in association with greater tumor dimension, higher pathological stage, higher depth of tumor invasion and lymphatic metastasis. Down-regulation of SPRY4-IT1 has been associated with poor prognosis of gastric cancer patients in this cohort (9).

Elevation of SPRY4-IT1 in patients with hepatocellular carcinoma has been associated with poor five year survival of patients. Besides, expression of SPRY4-IT1 in these patients has been correlated with TNM stage (21). **Table 3** summarizes the role of SPRY4-IT1 in cancers based on clinical studies.

**Table 3:** Effect of SPRY4-IT1 in cancers based on clinical studies.

Tumor type	Samples	Expression(Tumor *vs.* Normal)	Kaplan-Meier analysis (impact of SPRY4-IT1 up-regulation)	Univariate/Multivariate cox regression	Association of SPRY4-IT1 expression with Clinicopathologic characteristics	Reference
Colorectal cancer (CRC)	72 CRC tissues and normal tissues	up	poorer OS	_	_	(3)
	106 CRC tissues and ANCTs	up	poorer OS	SPRY4-IT1 levels are independent factors for CRC prognosis.	tumor bulk, depth of invasion, lymph node positivity, distant invasion, and tumor stage	(23)
	96 pair of CRC tissues and ANCTs	up	_	_	tumor size	(34)
	84 pair of CRC tissues and ANCTs	up	worse OS	SPRY4-IT1 level is an independent prognostic indicator for OS.	advanced TNM stage, depth of invasion, and metastasis	(24)
	88 CRC serum samples and 98 healthy controls	up	_	_	_	
	88 pair of CRC tissues and ANCTs	up	_	_	tumor size	(4)
	113 CRC tissues	up	shorter OS and DFS	_	lymph node metastasis and advanced-stage disease	(5)
Breast cancer	101 breast cancer patients	up	worse OS and DFS	_	_	(6)
	TCGA analysis:	up in patients with CD44+/CD24‐	_	_	_	
	102 pairs of tumor tissues and ANCTs	up	poorer OS and DFS	SPRY4-IT1 level is an independent prognostic factor for both OS and DFS.	large tumor size, high TNM stage, and lymph node metastasis	(35)
	48 pairs of tumor tissues and ANCTs	up	_	_	larger tumors with a higher tumor burden, and more advanced tumors	(7)
	101 breast cancer tissues	up	shorter OS and DFS	_	lymph node metastasis and advanced stage	(5)
Ovarian cancer	96 ovarian cancer tissues	up	shorter OS and DFS	_	lymph node metastasis and advanced-stage disease	(5)
	15 pairs of tumor tissues and ANCTs	down	higher OS and DFS	_	_	(26)
Gastric cancer (GC)	68 pairs of GC tissues and ANCTs	up	_	_	larger tumor size and advanced TNM stage	(8)
	61 pairs of GC tissues and ANCTs	down	higher OS and DFS	_	Decreased expression of SPRY4-IT1 is correlated with greater tumor bulk, advanced pathological stage, greater depth of invasion, and lymphatic metastasis.	(9)
Osteosarcoma	56 pairs of tumor tissues and ANCTs	up	_	_	metastases, recurrence, and tumor maximum diameter	(27)
Lung cancer	TCGA analysis: 412 LUAD patients	up	shorter OS	_	_	(13)
	88 pairs of tumor tissues and ANCTs	up	poorer prognosis	Levels of SPRY4-IT1 and histological grade were independent prognostic factors for OS.	larger tumor size, and high histological grade	
	121 pairs of NSCLC tissues and ANCTs	down	higher OS	Low levels of SPRY4-IT1 were independent predictors of poor survival for NSCLC.	tumor size, advanced pathological stage, and lymph node metastasis	(12)
Cervical cancer	100 pairs of cervical cancer tissues and ANCTs	up	shorter OS	Expression of SPRY4‐IT1 was an independent prognostic factor for OS of cervical cancer patients.	tumor size, FIGO stage, SCC‐Ag, and lymph node metastasis	(36)
Testicular germ cell tumor (TGCT)	13 TGCTs and 11 normal testis samples	up	_	_	_	(15)
Melanoma	70 cases of malignant melanoma and 79 normal controls	up	poorer prognosis	SPRY4‐IT1 was found to be an independent prognostic factor for OS in patients.	tumor site and TNM stage	(37)
Glioma	64 glioma specimens and 9 normal brain tissue specimens	up	_	_	_	(17)
	163 glioma tissues and ANCTs	up	poorer OS	Expression of SPRY4-IT1 and WHO grade were independently significant prognostic factors.	WHO grade, and tumor size	(38)
	18 pairs of glioma tissues and ANCTs	up	_	_	_	(18)
Pancreatic ductal adenocarcinoma (PDAC)	46 pairs of PDAC tissues and ANCTs	up	worse 5-year OS	SPRY4-IT1 was an independent predictor of poor OS.	advanced tumor stages and poor differentiation grade	(28)
Cholangiocarcinoma (CCA)	70 pairs of CCA tissues and ANCTs	up	worse OS and PFS	SPRY4-IT1 was an independent predictor of poor PFS and OS.	late tumor stage and advanced TNM stage	(29)
Gallbladder carcinoma (GBC)	38 pairs of GBC tissues and ANCTs	up	_	_	tumor sizes and tumor status, lymph node metastasis	(30)
Bladder cancer	60 pairs of bladder cancer tissues and ANCTs	up	_	_	high tumor grade, lymph node involvement and distant metastasis	(20)
	68 pairs of UCB tissues and ANCTs	up	shorter OS	Expression of SPRY4-1T1, histological grade, cancer stage and lymph node involvement were found to be independent prognostic factors for patients with UCB.	advanced tumor stage, higher histological grade, and positive lymph node metastasis	(31)
Hepatocellular carcinoma (HCC)	87 pairs of HCC tissues and ANCTs	up	_	_	differentiation, tumor size, and TNM stage	(39)
	Plasma of 60 HCC cases, 85 hepatitis B and cirrhosis patients, and 63 controls	higher in pre-operation than that at post-operation, hepatitis B and cirrhosis, and the control groups	_	_	_	
	82 pairs of HCC tissues and ANCTs	up	poor 5-year OS rate		TNM stage and metastasis	(21)
Esophageal squamous cell carcinoma (ESCC)	92 pairs of ESCC tissues and ANCTs	up	shorter OS	SPRY4-IT1 expression, lymph node metastasis, and TNM stage were found to be independent prognostic factors for OS of ESCC patients.	tumor differentiation, T classification, lymph node involvement, and clinical stage	(32)
	50 pairs of ESCC tissues and ANCTs	up	_	_	advanced clinical stages	(22)
	48 pairs of ESCC tissues and ANCTs	up	_	_	_	(40)
	plasma of 24 clinical samples and 24 normal controls	up	_	_	_	
	92 pairs of esophageal cancer tissues and ANCTs	up	shorter OS and PFS	_	tumor differentiation, T classification, lymph node metastasis, and pathological stage	(41)
Clear cell renal cell carcinoma (ccRCC)	98 pairs of ccRCC tissues and ANCTs	up	shorter OS	SPRY4-IT1 expression histological grade, tumor stage, lymph node metastasis and distant metastasis were found to be independent prognostic factors for OS of ccRCC patients.	histological grade, tumor stage, lymph node metastasis, and distant metastasis	(33)

ANCTs, adjacent non-cancerous tissues; OS, Overall survival; TNM, tumor‐node‐metastasis; DFS, disease-free survival; LUAD, lung adenocarcinoma; NSCLC, non-small-cell lung cancer; PFS, progression free survival.

Expression of SPRY4-IT1 in tissues and peripheral blood might be used for separation of healthy tissues/blood samples from those obtained from patients with neoplastic conditions (**Table 4**).

**Table 4:** Impact of SPRY4-IT1 in cancer diagnosis.

Tumor Type	Samples	Distinguishing potential	Area Under Curve	Sensitivity (%)	Specificity (%)	Accuracy (%)	References
Cervical cancer (CC)	100 pairs of CC tissues and ANCTs	cervical cancer tissues *vs* ANCTs	0.741	78.3	63.6	_	(36)
Melanoma	70 cases of malignant melanoma and 79 normal subjects	patients with malignant melanoma *vs* healthy controls	0.813	72.2	82.4	_	(37)
Esophageal squamous cell carcinoma (ESCC)	147 ESCC patients and 123 healthy controls	ESCC patients *vs* healthy controls	0.800	48.2	_	_	(40)

ANCTs, adjacent non-cancerous tissues.

## Discussion

SPRY4-IT1 has oncogenic roles in diverse tissues. A possible path of participation of SPRY4-IT1 in the carcinogenesis is through decreasing bioavailability of miRNAs such as miR-101-3p, miR‐6882‐3p and miR-22-3p. The sponging effect of SPRY4-IT1 on miR-101 has been verified in colorectal cancer, osteosarcoma, cervical cancer, bladder cancer, gastric cancer and cholangiocarcinoma. Thus, this miRNA is the main target of SPRY4-IT1 in the carcinogenesis process. In spite of the bulk of evidence pointing to the oncogenic roles of SPRY4-IT1 in diverse tissues, single studies in lung, ovarian and gastric cancers have reported a tumor suppressor role for this lncRNA. Notably, in gastric cancer, animal studies have also shown contradictory results. The number of passages of the cancer cell lines and other *in vitro* and *in vivo* conditions should be compared between these studies to find the underlying causes of such inconsistent results.

SPRY4-IT1 has functional interactions with HIF-1α, NF-κB/p65, AMPK, ZEB1, MAPK and PI3K/Akt signaling, thus it can influence the carcinogenesis from different aspects.

Diagnostic value of SPRY4-IT1 has been assessed in cervical malignancy, melanoma and esophageal squamous cell carcinoma, with the best values being reported in the melanoma. Since this lncRNA has been identified in serum exosomes of patients with cancer, it represents a possible candidate in non-invasive diagnostic strategies. Yet, these results should be confirmed in large cohorts of patients with different stages of cancers to appraise this potential application.

Except for three types of cancers, namely lung, ovarian and gastric cancers which have contradictory results, elevation of SPRY4-IT1 in other types of cancers has been associated with poor prognosis of patients.

Cumulatively, SPRY4-IT1 is a potential cancer-related lncRNA which can be used as a possible therapeutic target for diverse malignancies. Several issues should be solved before application of SPRY4-IT1-targeting strategies in the clinical setting the most important one being the possible tissue-specific effect of this lncRNA in the carcinogenesis. Moreover, the impact of genetic variants within *SPRY4-IT1* coding gene on susceptibility to cancer and response to therapeutic options should be appraised in future investigations.

## Author Contributions

SG-F wrote the draft and revised it. MT designed and supervised the study. SS and TK collected the data and designed the figures and tables. All authors contributed to the article and approved the submitted version.

## Conflict of Interest

The authors declare that the research was conducted in the absence of any commercial or financial relationships that could be construed as a potential conflict of interest.

## Publisher’s Note

All claims expressed in this article are solely those of the authors and do not necessarily represent those of their affiliated organizations, or those of the publisher, the editors and the reviewers. Any product that may be evaluated in this article, or claim that may be made by its manufacturer, is not guaranteed or endorsed by the publisher.
